# The ‘hub’ model for colorectal surgery: a viable paradigm shift?

**DOI:** 10.1308/rcsann.2024.0003

**Published:** 2025-04-08

**Authors:** P Janardhanan, A Khalid, MH Anwaar, R Williams, E Timms, S Ward, S Karandikar, M Dattani

**Affiliations:** University Hospitals Birmingham NHS Foundation Trust, UK

**Keywords:** Colorectal surgery, COVID-19, Hub, New Deal

## Abstract

**Introduction:**

Nationally, in the aftermath of the first COVID-19 lockdown, the waiting list for elective surgery is approximately 7 million. To ameliorate an evolving crisis and improve system resilience, the Royal College of Surgeons of England proposed a ‘New Deal for Surgery’, promoting COVID-light sites and elective hubs. We evaluate the short-term outcomes, safety and sustainability of the hub model at a large National Health Service trust.

**Methods:**

All major elective colorectal operations performed at the hub between 8 March 2021 and 8 March 2022 were included for analysis. Pertinent data on patient demographics, operative performance and postoperative outcomes were analysed using SPSS 27.

**Results:**

In total, 401 cases were analysed. There was one same-day cancellation because of the unavailability of beds (0.2%). Median distance displacement for patients for their primary surgery was +3.2 miles. Twenty-one patients (5.2%) required postoperative blood transfusion. One patient had nosocomial COVID-19 (0.2%), severe complications of Clavien–Dindo grade ≥3 were observed in 33 patient (8.2%) and transfer-out for higher level care occurred in 34 cases (8.5%). Forty-six 30-day readmissions (11.5%) and two deaths (0.4%) were noted. Median length of stay was 6 days.

**Conclusions:**

The volume of major colorectal surgery at the hub, with acceptable incidence of major complication, transfer-out and minimal patient displacement, attests to the efficacy and safety of the new model.

## Introduction

The SARS-CoV-2 (COVID-19) pandemic has resulted in the reorganisation of surgical service provision and practice on a global scale.^1^ The 3-month pause in elective surgery in 2020 increased the backlog of elective operations from 4.4 million prepandemic to an estimated 6 million in 2022, and 7 million by 2023.^2–5^ The growing backlog of surgery called for the development of surgical ‘hubs’ to provide exclusive elective services, focusing both on ‘high-volume, low-complexity’ and complex surgeries.^5–7^

**Figure 1:**
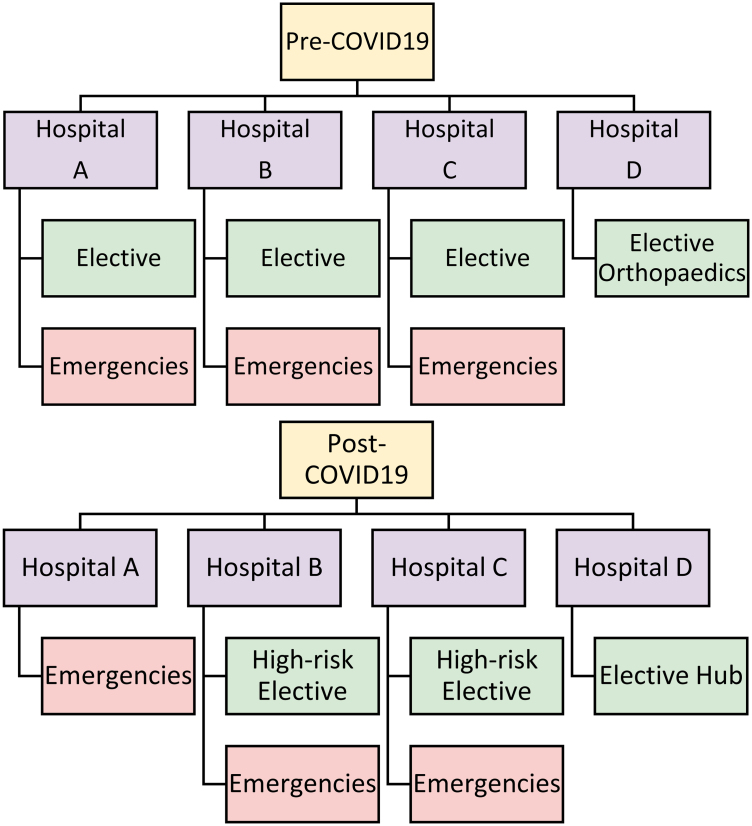
Overview of National Health Service trust service reconfiguration before and after COVID-19

Before the COVID-19 pandemic, elective and emergency general surgical services across one of the largest National Health Service (NHS) trusts in the UK were devolved to its component hospitals (henceforth referred to interchangeably as parent sites) across three sites: hospitals A, B and C. A fourth site affiliated with the trust, hospital D, had been used as a satellite site for medical admissions and elective orthopaedic surgery (Figure 1).

In line with proposals outlined by the Royal College of Surgeons England (RCS England) to improve the resilience of surgical units, general surgical services were reorganised within the trust to resume elective operations with minimal pandemic-related disruption.^8^ The new model saw hospital D as the designated ‘green’ site for most elective general surgery, urology, and ear, nose and throat surgeries. Parent hospitals were mostly used for emergency surgery.

**Figure 2:**
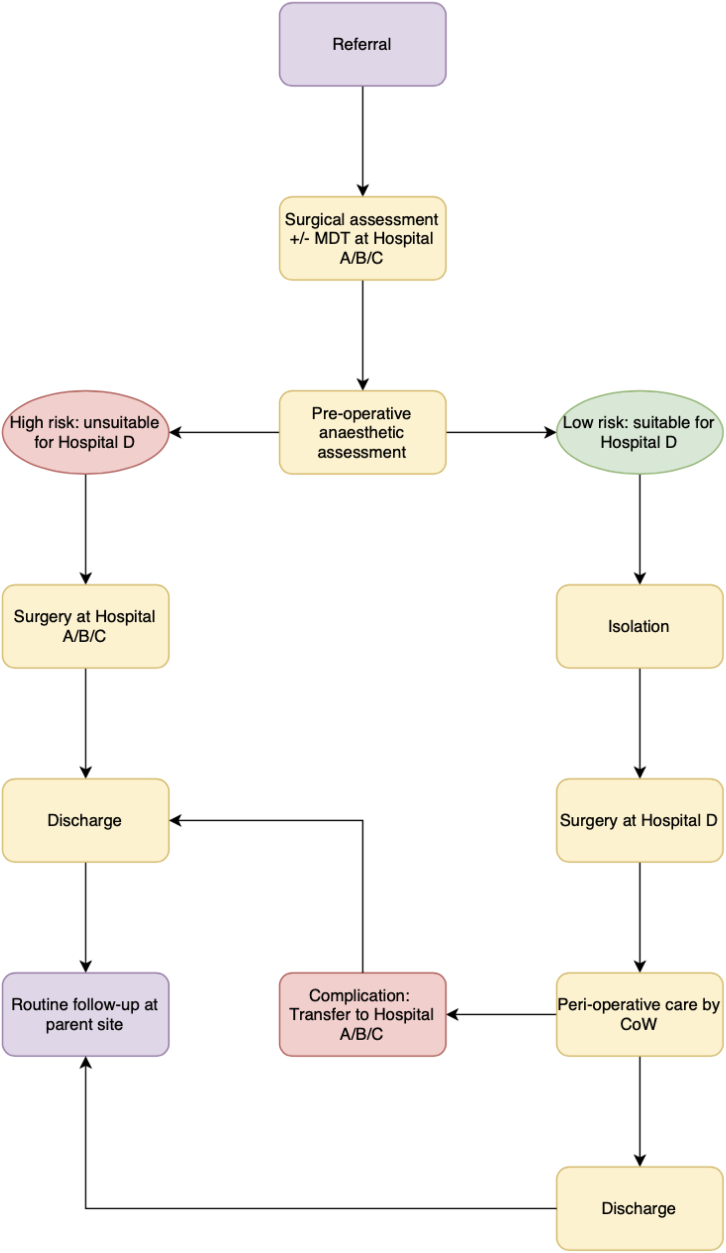
Hub pathway

After service reorganisation, new referrals, including 2-week wait, were assessed in an outpatient clinic and investigated at either the constituent hospitals or hospital D. New cancer diagnoses were discussed in devolved multidisciplinary team (MDT) meetings at parent sites (as an aside, devolved MDTs existed separately for inflammatory bowel disease). Patients were counselled on their diagnosis on an outpatient basis at parent sites or hospital D by the allocated consultant surgeon, with input from colorectal nurse specialists. Those requiring surgery underwent anaesthetic assessment at a parent hospital to gauge their suitability for surgery at hospital D. A formalised prehabilitation programme was not set up at the time of writing. If suitable for surgery in hospital D, cases were listed via a centralised booking system. Two operating theatres functioning with a two-session daily capacity between Monday and Friday were allocated for colorectal surgery at hospital D, and lists were allocated equally among surgeons from the constituent hospitals. Patients added to elective lists at the parent sites during or before the pandemic were assimilated into the hospital D elective pool if suitable (Figure 2). Broadly, surgeons operated either at affiliated parent sites or hospital D.

**Figure 3:**
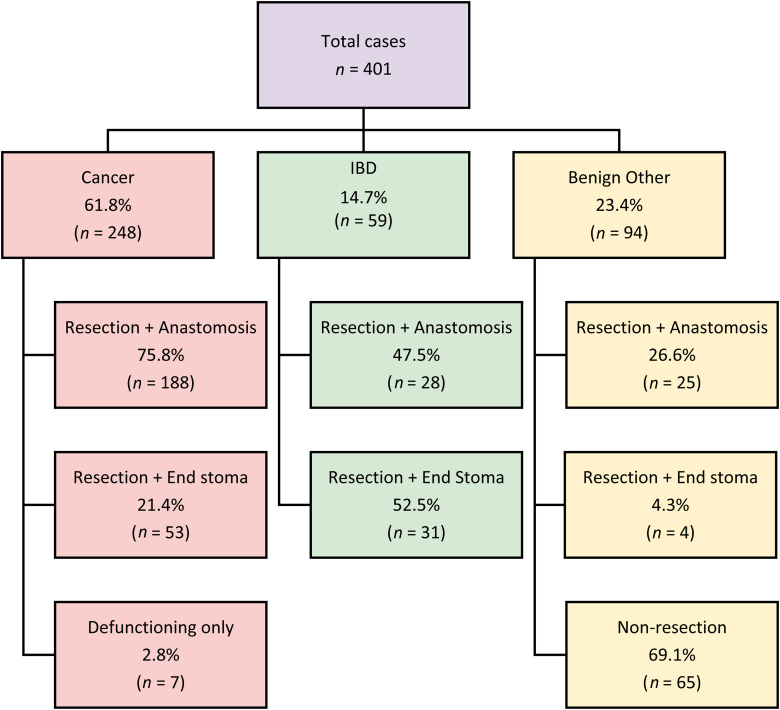
Schema for resections and anastomosis based on case type

Appropriate patient selection for surgical intervention at hospital D was of paramount importance, because this site did not have an intensive care unit (ICU), out-of-hours blood bank, interventional radiology or provision for parenteral nutrition. Clear criteria for operative suitability at hospital D incorporating risk stratification were established to ensure appropriate patient selection, the details of which are beyond the scope of this paper.

An enhanced postoperative care (EPOC) unit was designed within hospital D for the provision of basic postoperative respiratory and cardiovascular support. The EPOC was staffed by on-site intensive care doctors. Preoperatively, if patients were considered to require critical care input, surgery was facilitated at the parent sites. Because of the large patient population and the large size of the geographical catchment covered by the trust, it was deemed prudent to offer surgery for ‘high-risk’ patients in multiple sites.

Postoperatively, a designated colorectal ‘consultant of the week’ (CoW) oversaw daily patient progress; the consultant on-call rota was modified to 1:24 additional cover across the parent sites to account for CoW responsibilities. The CoW provided 24h cover (off-site outside normal working hours) for the week, with support from two or three Foundation Year 1 (FY1) level doctors and one FY2 level doctor. Redistribution of surgical nursing and up-skilling auxiliary staff permitted provision of postoperative patient care in a repurposed manner. The principles of enhanced recovery after surgery were applied to postoperative patient care.

In the event of physiological deterioration requiring ICU input (advanced single or multiple organ support), a major unplanned reoperation, requirement for parenteral nutrition or interventional radiology, patients were transferred to the parent institution after liaison with respective on-call surgical and/or ICU teams. No provisions for routine out-of-hours access to theatres at hospital D exist at the time of writing, thus necessitating transfer-out for salvage surgery or reintervention. If the patient was deemed to require a higher level of care by EPOC intensivists, urgent transfer was arranged to any of the constituent hospitals with ICU capacity, with continuity of surgical care being delivered by colorectal surgeons at the receiving hospital. This model has been operational since resumption of elective general surgery on 8 March 2021, and has now become the new way of working.

### Aims and objectives

The primary aim of this study was to evaluate the short-term outcomes, safety and sustainability of the hub model for elective colorectal surgery at a large NHS trust. As a secondary aim, the impact of travel distance for patients was studied: for this model to be acceptable to patients, travel disruptions must be minimal.^9^

## Methods

### Inclusion/exclusion

All major elective colorectal operations performed at hospital D between 8 March 2021 and 8 March 2022 were included. Prospectively collated data from operative logs and electronic patient records were analysed retrospectively. The evaluated outcome indices are complication, hub–parent hospital transfer, 90-day mortality, 30-day readmission and nosocomial COVID incidence. Diagnostic laparoscopy, endoscopic procedures under general anaesthetic and day case procedures were excluded from further analysis. Procedures included in the analysis are categorised in Table 1.

**Table 1:** Categorisation of colorectal operations

Operative category	Sub-category	Operations included
Resection	Small bowel	Any small bowel resection distal to ligament of Treitz
	Right-sided colonic	Ileocolic resection; right hemicolectomy; extended right hemicolectomy
	Left-sided colonic	Left hemicolectomy; sigmoid colectomy
	Rectum	Anterior resection; abdominoperineal excision of rectum; proctectomy; pelvic exenteration
	Other	Subtotal colectomy; panproctocolectomy; cytoreduction + HIPEC
Non-resection	Primary defunctioning	Formation of ileostomy or colostomy without resection
	Reversal of stoma	Reversal of ileostomy or colostomy
	Other	Incisional hernia repair; parastomal hernia repair

HIPEC = Hyperthermic intraperitoneal chemotherapy

**Table 2:** Displacement analysis with comparison between subgroups based on patient’s parent hospital

	Distance to Hospital D (miles)			Overall displacement (miles)		
	Median	IQR	Range	Median	IQR	Range
Pooled patients	8.2	4.5 to 17.3	0.3 to 143.0	3.2	−1.7 to 8.2	−16.7 to 22.7
Hospital A patients	4.8	3.2 to 7.4	0.4 to 80.7	0.9	−3.1 to 3.5	−16.2 to 16.4
Hospital B patients	17.2	11.0 to 20.7	0.7 to 97.3	12.2	4.3 to 14.8	−16.7 to 18.2
Hospital C patients	10.5	6.2 to 17.5	1.2 to 135.0	5.8	0.2 to 8.4	−14.9 to 22.7

IQR = interquartile range

### Definitions

Underlying pathology was coded into three categories: neoplasia (adenocarcinoma, malignant polyps and high-grade dysplastic lesions); inflammatory bowel disease (IBD); and other benign conditions. For cancer resections, the location of the lesion was categorised into: small bowel; right colon (caecum, ascending and proximal half of transverse colon); left colon (distal half of transverse colon, descending and sigmoid colon); or rectum. Major complications were defined in accordance with the Clavien–Dindo classification; those graded 3 and above were included for further analysis.^10^

Distance displacement to gauge travel disruption for patients was calculated using Google Maps. The shortest distances from a patient’s usual residence and parent hospital, and from the patient’s usual residence to hospital D were used to calculate overall displacement using the formula:

$$\begin{array}{rl} & \mathrm{Overalldisplacement}=(\mathrm{Shortest}\;\mathrm{distance}\;\mathrm{from}\;\mathrm{patient}\;\\ & \mathrm{home}\;\mathrm{to}\;\mathrm{Hospital}\;D)\text{–}(\mathrm{Shortest}\;\mathrm{distance}\;\mathrm{from}\;\mathrm{patient}\;\\ & \mathrm{home}\;\mathrm{to}\;\mathrm{parent}\;\mathrm{hospital}) \end{array}$$

## Results

### Demographics

A total of 401 cases met the inclusion criteria for further analysis (Figure 3). The median age of patients was 65 years (interquartile range [IQR] 51.5–73 years; range 18–90 years). Some 211 patients were male (52.6%) and 190 were female (47.4%). The median BMI of the cohort was 27 (IQR 23.3–31; range 13–52) and the median ASA grading was 2 (IQR 2–2; range 1–4). All patients tested negative for COVID-19 preoperatively. There was one (0.2%) cancellation on the day of surgery because of a lack of EPOC capacity.

In terms of displacement, it is first prudent to consider the distance to hospital D from each of the parent sites. The shortest distances from hospitals A, B and C to hospital D are 6.3, 17.4 and 9.2 miles, respectively. Median total displacement to hospital D was +3.2 miles (IQR −1.7 to 8.2 miles; range −16.7 to 22.7 miles) (Table 2).

### Procedure-related parameters

**Figure 4:**
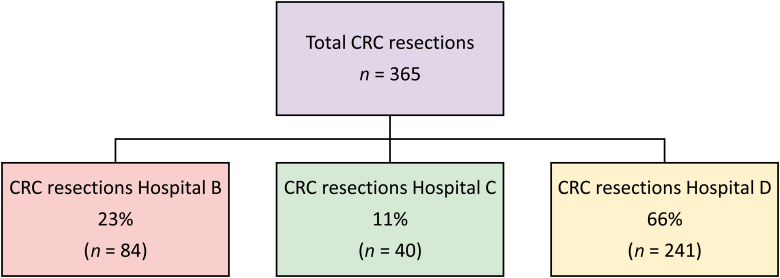
Breakdown of colorectal cancer resections across the trust

A summary of procedures is provided in Table 3. Most cases were performed for treatment of cancer (248; 61.8%) and the remainder for benign pathology. In terms of overall volume of colorectal cancer resections, 365 resections were performed across the trust, of which 241 were performed at hospital D (Figure 4). Of the non-cancer operations, 59 cases (14.7%) were performed for IBD and the remaining 94 (23.4%) for other conditions. Rectal cancer accounted for 95 cancer cases (38.3%), followed closely by right-sided cancers in 78 cases (31.5%) and left-sided cancers in 70 cases (28.2%); small bowel cancers accounted for a small subset of 5 cases (2.0%).

**Table 3:** Summary of operations

Operative category	Sub-category	Percentage of total cases (*n*)
Resections (*n* = 329, 82.0%)	Small bowel	2.0 (8)
	Right-sided colonic	27.7 (111)
	Left-sided colonic	3.5 (30)
	Rectum	35.2 (141)
	Other	9.7 (39)
Non-resections (*n* = 72, 18.0%)	Primary defunctioning	2.2 (9)
	Reversal of stoma	9.5 (38)
	Other	6.2 (25)

Some 329 (82.0%) procedures were resections, of which 278 (84.5%) cases underwent primary anastomosis. There were 135 (33.7%) operations involving the formation of new stomata, 55 (37.8%) of which were end colostomies and 39 (28.9%) were covering loop ileostomies. In resections, surgical access was laparoscopic in 213 cases (64.7%), whereas 116 (35.3%) were open procedures. Within the subset of laparoscopic procedures, 29 underwent open conversion. Of the cancer cases, access was laparoscopic in 166 cases (66.9%) and primarily open in 82 (33.1%). Open conversion from the initial laparoscopic approach occurred in 19 cancer cases.

### Postoperative outcomes

Of the 401 procedures, 318 patients (79.3%) were transferred to EPOC postoperatively, with the remaining 83 patients (20.7%) receiving ward-level care directly. Twenty-one patients (5.2%) required transfusions in the postoperative period, and complications graded ≥3 on the Clavien–Dindo classification were observed in 33 cases (8.2%). One case (0.2%) of nosocomial COVID-19 confirmed by polymerase chain reaction was noted, although the validity of this remains contentious, because the patient had tested positive in the weeks preceding surgery.

Transfer back to the parent site because of complications or protracted recovery occurred in 34 cases (8.5%), details of which are given in Table 4. Total median length of stay (LOS), including time spent in the parent institution if transferred out, was 6 days (IQR 4–9 days; range 1–80 days). Interestingly, median LOS was also 6 days (IQR 4–8 days; range 1–23 days) when cases that were transferred out were excluded from the analysis. We observed forty-six 30-day readmissions (11.5%) during this period, and two deaths within 90 days (0.4%): one in hospital D at the index admission because of a major myocardial infarction, and the second postdischarge, presumed to be caused by a myocardial infarction.

**Table 4:** Summary of transfers out and 30-day readmissions

		Percentage of total cases (*n*)
Reason for transfer-out (*n* = 34, 8.5%) 3 missing cases	Cardiac support	11.8 (4)
	Respiratory support	5.9 (2)
	Nutritional support	26.5 (9)
	Surgical reintervention	23.5 (8)
	Multiple	23.5 (8)
Reason for readmission (*n* = 46, 11.5%) 7 missing cases	Medical	32.6 (15)
	Postoperative infection	21.7 (10)
	Bowel obstruction	13.0 (6)
	Stoma-related	17.4 (8)

## Discussion

The COVID-19 pandemic has necessitated a reconfiguration of surgical service provision to increase resilience and robustness against future disruptions related to pandemic conditions. RCS England outlined a 12-point plan in ‘New Deal for Surgery’ to facilitate this process. A key recommendation was the creation of ‘surgical hubs’ and ‘COVID-light sites’.^6,8^ Although the hub-and-spoke model has been adopted by specialties such as vascular, hepatopancreatobiliary and resectional upper gastrointestinal surgery, the concept is relatively uncommon in colorectal surgery. Here, we report on the experience of colorectal surgery after adoption of the hub model at one of the largest NHS trusts, with the aims of evaluating the short-term outcomes, safety and sustainability of the model.

Addressing the efficacy of the model, our data suggest that the hub model is effective in the delivery of elective colorectal surgery and limiting nosocomial COVID-19 infections: 401 procedures in a 1-year period with only 1 (0.2%) case of nosocomial COVID-19. This was an asymptomatic patient who had previously contracted the virus in the community in the preceding 4 weeks leading up to surgery and was therefore thought to be shedding the virus. Our findings support the notion that both open and laparoscopic surgery can be safely performed for malignant and benign pathologies in the hub. Same-day cancellation because of a lack of bed capacity was negligible at 0.2%, which is arguably a reliable indicator of system robustness.

Looking more closely at colorectal cancers, the performance is comparable with the national average, as declared in the National Bowel Cancer Audit (NBOCA) Annual Report 2021 for proportion of cases performed laparoscopically, LOS, 30-day readmission and overall survival, and outperforms in the rates of ileostomy formation in rectal cancer.^11^ This attests to the efficacy of the hub model with the caveat being the heterogeneity of the NBOCA cohort and the homogeneity of our hub cohort. As shown in Table 5, comparison of outcomes from the hub cohort and pooled outcomes across the trust reveals only marginal improvement in 30-day unplanned readmissions and 90-day survival attributable to patient selection. Unfortunately, we are unable to comment on the impact of patient selection on stoma formation and LOS because these data are currently not available for analysis.

**Table 5:** Comparison against NBOCA 2021 performance

	National (%)	Hospital D cancers (*n* = 241)*
Laparoscopic surgery	63	66.8 (161)
Loop ileostomy in rectal cancers	30	27.2 (25)
End colostomy in rectal cancers	38	39.1 (36)
Length of stay	6 days	6 days (IQR 5–9)
30-day unplanned readmission	11	8.3 (20)
90-day survival	98	99.2 (239)

*Unless otherwise stated values are given as % (*n*)

NBOCA = National Bowel Cancer Audit

In evaluating the safety of the system, we use rates of transfer-out, complications, nosocomial COVID-19 and mortality as objective outcome measures. Major colorectal cancer resections carry significant risk: elective resections carry 90-day mortality risk of 1.6%, which increases to 5.1% in urgent resections.^11^ In our cohort 90-day mortality stands at 0.8%; although this appears to be superior to the national average of 1.6%, we accept that the patients undergoing surgery at the hub are highly selective, in the low–moderate risk category. Recently Carvalho *et al* published their experience of colorectal surgery with complication rate of 4.16%.^12^ This is better than the rate observed at our institution at 8.2%, although our sample size was more than double that of Carvalho *et al*.

The hub model has demonstrated safety by achieving comparable outcomes despite limited resources (blood bank, interventional radiology, nutrition and critical care facilities). For patients requiring higher level care, transfer-out to the other hospitals generally occurred within 24h, and much more expediently as dictated by the clinical condition of the patient. Transfer of patient care between sites was facilitated by bed managers, and timely communication between surgical and ITU colleagues across sites. If on-site service provision at the hub is improved, this can mitigate transfer-out: for instance, the availability of on-site nutritional support can reduce 25% of transfers. Given the lack of standards or direct comparators for performance, major complication and transfer-out rates under 10% seem acceptable, especially when considering the accepted morbidity associated with major colorectal resections.

The assessment of sustainability of the hub model is perhaps most challenging, because numerous factors contribute to it. In addition to the parameters analysed here, patient satisfaction, clinician satisfaction and financial implications all have an important role in predicting long-term viability. For the new model to be acceptable to patients, overall displacement needs to be minimised. In a systematic review of 57 studies evaluating healthcare utilisation across Europe, North America and South America, the authors observed fewer visits to specialists for treatment than to primary care physicians in patients from the lowest socio-economic strata.^9^ Potential barriers to specialist treatment in this group may be waiting times, cost and proximity to the treatment centre. Our data demonstrate that patients face minimal travel disruption because of service reconfiguration.

If distance displacement and on-the-day cancellation rates are used as proxy markers for patient satisfaction, the surety of scheduled surgery proceeding as planned and minimal overall displacement of an added 3.2 miles to receive care can suggest adequate patient satisfaction. Further work is needed to formally evaluate patient satisfaction, surgeon satisfaction and overall financial implications.

In the wider context of a paradigm shift to multicentre working, important consideration must be given to the effects of continuity of patient care on patient outcomes. Continuity of care been shown to affect the mortality rate following elective colorectal surgery.^13^ The establishment of a centralised electronic system in our trust for clinical documentation, reviewing patient observations and results that can be accessed remotely, enabled clinicians to monitor patient progress remotely. Although this measure alone did not overcome the challenges posed by multisite working, it certainly alleviated clinician apprehensions about monitoring postoperative patient progress.

A large study from the US comprising more than 20,000 patients demonstrated that readmission after elective colorectal resection carried 2.16-fold increase mortality if treated by a different surgeon at the index hospital, and 2.8-fold increase in mortality if treated at a different hospital and by a different surgeon. To mitigate this, we established clear protocols for patients requiring transfer or presenting for readmission. Patients being transferred out from hospital D back to parent institutions would be formally handed over to the on-call CoW, with an update to the index surgeon. Patients requiring readmission were advised to either liaise with the index surgeon directly, or to present to the parent institution in the event of an emergency.

Furthermore, proponents of single-site working may raise concerns about the impact of clinical outcomes when surgeons work across multiple sites. The assertion that surgeons working across several sites may compromise clinical outcomes has not been shown to influence the surgical mortality rates. In a large study from the US, multisite and single-site surgeons had equivalent outcomes,^14^ suggesting that multisite working practices are safe and feasible, provided robust mechanisms are in place to deliver postoperative care. Fry *et al* proposed that the greatest reductions in surgical mortality are achieved by dramatic reductions in failure-to-rescue rates (reduction in mortality after treatable complication), with only marginal gains from focusing on absolute reductions in serious complications.^15^ Round-the-clock availability of a consultant (CoW) at our hub with agreed pathways for transfer-out attests for early detection of complications, improved rescue rates, and low mortality.

Our work adds to the growing literature analysing the feasibility of the colorectal hub model, with specific focus on postoperative outcomes. Our hub data demonstrate that major elective colorectal surgery can be safely carried out with acceptable outcomes. This represents a paradigm shift in how we deliver not only colorectal surgery, but also wider elective surgical care that is resilient to unavoidable surges in external pressures, however transient their nature. For optimal performance, hubs require pathways to detect and manage complications, and to offer salvage in a timely and effective manner. This requires adequate foresight, planning, allocation of resources, restructuring of workforce plans and financial investment.

The hub model for colorectal surgery shows promise, but further work is needed to definitively gauge long-term sustainability. Specific to our trust, future directions to entertain are: the impact of service reorganisation on outcomes for ‘high-risk’ patients at parent sites; the feasibility of improving essential services at hospital D (that are currently unavailable) to mitigate transfers; and evaluating patient satisfaction with the model.

Nonetheless, we demonstrate here that hubs can be established to deliver major colorectal surgery in a safe and efficient manner, provided that robust measures for early detection and treatment of complications, contingencies for parenteral nutrition and interventional radiology are in place. Because our trust was able to achieve satisfactory clinical outcomes in the early period of hub inception – with relatively basic postoperative support – and the model is under continual appraisal, we believe that this model is replicable across large trusts in the UK to tackle the growing elective backlog. There has been unanimous acceptance of the hub model of working by colorectal surgeons and anaesthetists at our trust given minimal on-day cancellations, continuity of care measures and contingency pathways for unexpected patient deterioration. We firmly believe that the hub model shows promise in the campaign to tackle the growing elective surgery backlog, especially in the context of looming winter pressures, and should be implemented widely.

## Conclusion

The hub model for colorectal surgery facilitated the provision of elective surgery with minimal disruptions attributable to COVID-19 in a safe and sustainable manner.
